# Impact of Maternal Hyperglycemic and Hypertensive Disorders on Perinatal Outcomes Across the COVID-19 Pandemic

**DOI:** 10.1089/whr.2025.0019

**Published:** 2025-04-28

**Authors:** Lixia Zhang, Yun Shen, Ronald Horswell, Jessica Lin, San Chu, S. Amanda Dumas, Gang Hu

**Affiliations:** ^1^ Pennington Biomedical Research Center, Baton Rouge, Louisiana, USA.; ^2^ Department of Pediatrics, Louisiana State University Health Sciences Center, New Orleans, Louisiana, USA.

**Keywords:** hyperglycemic disorders, hypertensive disorders, COVID-19, adverse pregnancy outcomes

## Abstract

**Objective:**

This study aimed to evaluate the joint associations of maternal hyperglycemic and hypertensive disorders with adverse pregnancy outcomes across the coronavirus disease 2019 (COVID-19) pandemic.

**Methods:**

This retrospective study included 110,447 Louisiana Medicaid pregnant women with first-time delivery from January 1, 2016, to December 31, 2021. Associations between hyperglycemic as well as hypertensive disorders and adverse pregnancy outcomes in pregnancy during prepandemic, early pandemic, and late pandemic were assessed by binary logistic regression.

**Results:**

The odds ratios of above adverse pregnancy outcomes were significantly higher during the early and late COVID-19 pandemic than those before the pandemic. Maternal gestational diabetes mellitus and diabetes before pregnancy were associated with higher risks of preterm birth, primary cesarean section, large for gestational age (LGA), macrosomia, neonatal hypoglycemia, neonatal jaundice, and neonatal respiratory distress syndrome (NRDS; all *p* < 0.05), respectively, compared with women with normal glucose during pregnancy. Maternal gestational hypertension, preeclampsia or eclampsia, and pre-existing hypertension were associated with higher risks of preterm birth, primary cesarean section, low birth weight (exception for gestational hypertension), small for gestational age, LGA (exception for preeclampsia or eclampsia), macrosomia (exception for preeclampsia or eclampsia), neonatal hypoglycemia, neonatal jaundice, and NRDS (all *p* < 0.05), respectively, compared with women with normal blood pressure during pregnancy. Most of these associations during the early and late pandemic were consistent with those before the COVID-19 pandemic.

**Conclusions:**

Maternal hyperglycemic and hypertensive disorders during pregnancy, compared with maternal normal glucose or blood pressure during pregnancy, were associated with higher risks of adverse maternal and neonatal outcomes. Interventions should be taken to help individuals achieve glycemic and blood pressure control to decrease the risk of adverse perinatal outcomes regardless of the COVID-19 pandemic.

## Introduction 

Hyperglycemic disorders during pregnancy and hypertensive disorders of pregnancy (HDP) are the most common pregnancy complications worldwide due to increasing maternal age and higher prevalence of obesity as well as unhealthy lifestyle habits among women.^1,2^ Hyperglycemic disorders during pregnancy can arise from pre-existing conditions such as type 1 diabetes and type 2 diabetes to gestational diabetes mellitus (GDM), which emerges during pregnancy. HDP encompasses chronic hypertension that exists before pregnancy, gestational hypertension that develops during pregnancy, pre-eclampsia, and eclampsia. These maternal issues are associated with both immediate and long-term adverse outcomes, including increased risks of cesarean section, premature birth, low birth weight (LBW), excessive birth weight (macrosomia), newborn low blood sugar, respiratory problems in newborns, and higher risks of hypertension, diabetes, metabolic syndrome, and cardiovascular disease in the future.^3–10^ This presents a significant health challenge for both mothers and their children.

The coronavirus disease 2019 (COVID-19) pandemic, caused by the novel severe acute respiratory syndrome coronavirus 2, has heightened concern regarding its impact on maternal and neonatal health. Several observational studies and systematic reviews have investigated the pandemic’s impact on hyperglycemic disorders during pregnancy and HDP as well as adverse maternal and neonatal outcomes.^11–14^ These adverse outcomes include elevated risks of severe maternal morbidity, preterm birth, stillbirth, LBW, and potential neurodevelopmental challenges in offspring. However, another systematic review suggested that COVID-19 infection did not significantly affect pregnancy outcomes.^15^ This indicates that the full impact of COVID-19 on pregnancy outcomes remains unclear. Notably, there have been no studies exploring how COVID-19 affects the relationship between maternal hyperglycemic and hypertensive disorders and adverse pregnancy outcomes. The objectives of the present study were to assess the joint associations of maternal hyperglycemic and hypertensive disorders during pregnancy with adverse pregnancy outcomes across the COVID-19 pandemic in a comprehensive observational study utilizing data from the Louisiana Medicaid database.

## Methods

### Study design, population, and data source

The present study conducted a retrospective analysis of electronic health records from the Louisiana Medicaid dataset. Medicaid administrative data, collected by the Centers for Medicaid and Medicare Services, are primarily derived from reimbursement claims, reflecting the payment for services provided. These claims data are clinically reliable and encompass a broad array of essential health care attributes, including admission and discharge dates, diagnostic and procedure codes, source of care, and demographic information (such as date of birth, age, race and ethnicity, and sex).

**FIG. 1.**
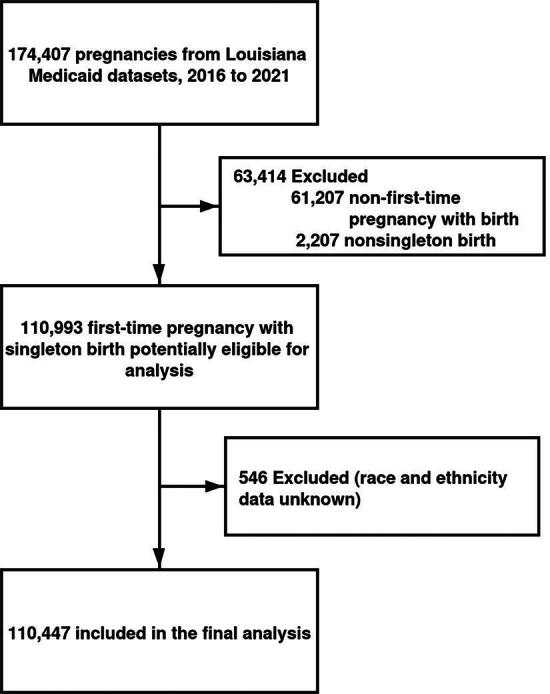
Participants flow chart.

Our analysis focused on women who gave birth between January 1, 2016, and December 31, 2021. We excluded records of nonfirst-time pregnancies, nonsingleton births, and cases with unknown race or ethnicity, culminating in a final sample of 110,447 pregnant women for our analysis (Fig. 1). Pregnancies were identified using the Tenth Revisions of the International Classification of Disease (ICD-10) code. The study and analytic plan were approved by the Institutional Review Boards at the Pennington Biomedical Research Center and the Louisiana Department of Health. The study followed the Strengthening the Reporting of Observational Studies in Epidemiology reporting guideline.

### Hyperglycemic and hypertensive disorders in pregnancy

Hyperglycemic and hypertensive disorders during pregnancy were identified using ICD-10 codes. GDM cases were identified by using the ICD-10 code O24.4, which represents “gestational diabetes mellitus.” Identification of diabetes that was present before pregnancy was done using the ICD-10 codes O24.0 and O24.1, which refer to pre-existing diabetes that occurred in pregnancy for types 1 and 2 diabetes, respectively. Participants during pregnancy were divided into three subgroups according to their glucose status: normal glucose, GDM, and diabetes before pregnancy. HDP included pre-existing chronic hypertension, gestational hypertension, pre-eclampsia, and eclampsia. Pre-existing chronic hypertension before pregnancy was identified by the ICD-10 code O10 that selected women who had elevated blood pressure predating the pregnancy and ICD-10 code O11 that selected women who had preeclampsia superimposed on chronic hypertension. Gestational hypertension was identified using the ICD-10 code O13 that specifically selected women who were diagnosed at or after 20 weeks’ gestation. Pre-eclampsia was identified by the ICD-10 code O14 that selected women who were diagnosed at or after 20 weeks’ gestation. Eclampsia was identified by the ICD-10 code O15. Participants in pregnancy were classified into four subgroups according to their blood pressure status: normal blood pressure, gestational hypertension, pre-eclampsia or eclampsia, and pre-existing hypertension.

### Definition of COVID-19 pandemic periods

The definition of COVID-19 pandemic periods was as follows: pregnancies impacted by the pandemic were those with deliveries after March 13, 2020, which marked the date of the first COVID-19 case reported in Louisiana. As a result, we established three groups based on delivery dates corresponding to the calendar years: the prepandemic period (from January 1, 2016, to March 12, 2020), the early pandemic period (from March 13, 2020, to March 12, 2021), and the late pandemic period (from March 13, 2021, to December 31, 2021). In addition, participants were further categorized into subgroups based on the presence of hyperglycemic and hypertensive disorders during pregnancy across different stages of the COVID-19 pandemic, with the specific numbers in Supplementary Table S1.

### Adverse pregnancy outcomes

Adverse pregnancy outcomes were identified using the ICD-10 codes. Maternal outcomes included primary cesarean section (ICD-10 codes O66.4, O75.82, and O82, except O34.21), which was defined as cesarean section performed to women with no previous history of cesarean delivery. Neonatal outcomes were preterm birth (birth at <37 gestational weeks, ICD-10 codes O60.1 and P07.3), LBW (birth weight <2,500 g, ICD-10 codes P07.0 and P07.1), macrosomia (birth weight ≥4,000 g, ICD-10 codes P08.0 and P08.1), small for gestational age (SGA, birth weight <10^th^ percentile for a given gestational age, ICD-10 codes P05.0 and P05.1), large for gestational age (LGA, birth weight >90^th^ percentile for a given gestational age, ICD-10 code P08.1), neonatal hypoglycemia (ICD-10 codes P70.0, P70.1, and P70.4), neonatal jaundice (ICD-10 code P59), and neonatal respiratory distress syndrome (NRDS, ICD-10 code P22).

### Statistical analysis

One-way analysis of variance and Chi-square tests were used to identify any differences in the characteristics of the women in the study and the prevalence of adverse pregnancy outcomes according to the COVID-19 pandemic status. We applied binary logistic regression models to assess the single and joint effects of different levels of maternal hyperglycemic or hypertensive disorders during pregnancy and the COVID-19 pandemic on adverse maternal–perinatal outcomes. All analyses were adjusted for maternal age, race, and ethnicity. We reported adjusted odds ratios (ORs) along with 95% confidence intervals (CIs) to delineate the relationship between predictor and outcome variables. A *p* value of <0.05 was considered statistically significant. All statistical analyses were performed using R software, version 4.3.2.

## Results

### Effect of the COVID-19 pandemic on adverse pregnancy outcomes

The final analysis included 110,447 participants, among whom 82,266 delivered prepandemic, 16,305 were exposed to the early stages of the COVID-19 pandemic, and 11,876 to the late stages. Over these periods, there were minor shifts in maternal age, race, and ethnicity (all *p* < 0.001), as shown in Table 1. Deliveries during both the early and late stages of the pandemic compared with deliveries during prepandemic showed a significantly higher prevalence of hyperglycemic disorders during pregnancy, HDP, primary cesarean sections, LBW, SGA, LGA, macrosomia, neonatal hypoglycemia, neonatal jaundice, and NRDS (all *p* < 0.001), along with a slightly lower incidence of preterm birth (*p* = 0.03).

**Table 1.** Characteristics and Adverse Pregnancy Outcomes of Participants Stratified by the COVID-19 Pandemic

Variables	Total	COVID-19 pandemic^a^			*p* value^b^
		**Prepandemic**	**Early Pandemic**	**Late pandemic**	
No. of participants	110,447	82,266	16,305	11,876	
Age, mean (SD), years	26.2 (6.04)	26.3 (5.97)	25.9 (6.25)	25.7 (6.19)	<0.001
Race and ethnicity, *n* (%)^c^					<0.001
White	48,385 (43.8)	37,074 (45.1)	6,481 (39.7)	4,830 (40.7)	
Black	44,255 (40.1)	32,858 (39.9)	6,705 (41.1)	4,692 (39.5)	
Other^c^	17,807 (16.1)	12,334 (15.0)	3,119 (19.1)	2,354 (19.8)	
Diabetes during pregnancy, *n* (%)					<0.001
Normal glucose	100,180 (90.7)	74,934 (91.1)	14,537 (89.2)	10,709 (90.2)	
Gestational diabetes mellitus	7,357 (6.66)	5,191 (6.31)	1,338 (8.21)	828 (6.97)	
Diabetes before pregnancy	2,910 (2.63)	2,141 (2.60)	430 (2.64)	339 (2.85)	
Hypertensive disorders of pregnancy, *n* (%)					<0.001
No	84,808 (76.8)	65,237 (79.3)	12,283 (75.3)	8,798 (74.1)	
Yes	25,639 (23.2)	17,029 (20.7)	4,022 (24.7)	3,078 (25.9)	
Preterm birth, *n* (%)					0.03
No	102,104 (92.4)	75,959 (92.3)	15,150 (92.9)	10,995 (92.6)	
Yes	8,343 (7.55)	6,307 (7.67)	1,155 (7.08)	881 (7.42)	
Primary cesarean section, *n* (%)					<0.001
No	100,049 (90.6)	74,777 (90.9)	14,668 (90.0)	10,604 (89.3)	
Yes	10,398 (9.41)	7,489 (9.10)	1,637 (10.0)	1,272 (10.7)	
Low birth weight, *n* (%)					<0.001
No	103,144 (93.4)	76,985 (93.6)	15,121 (92.7)	11,038 (92.9)	
Yes	7,303 (6.61)	5,281 (6.42)	1,184 (7.26)	838 (7.06)	
Small for gestational age, *n* (%)					<0.001
No	105,061 (95.1)	78,893 (95.9)	15,112 (92.7)	11,056 (93.1)	
Yes	5,386 (4.88)	3,373 (4.10)	1,193 (7.32)	820 (6.90)	
Large for gestational age, *n* (%)					<0.001
No	107,008 (96.9)	79,837 (97.0)	15,686 (96.2)	11,485 (96.7)	
Yes	3,439 (3.11)	2,429 (2.95)	619 (3.80)	391 (3.29)	
Macrosomia, *n* (%)					<0.001
No	106,853 (96.7)	79,719 (96.9)	15,665 (96.1)	11,469 (96.6)	
Yes	3,594 (3.25)	2,547 (3.10)	640 (3.93)	407 (3.43)	
Neonatal hypoglycemia, *n* (%)					<0.001
No	102,880 (93.1)	77,101 (93.7)	14,905 (91.4)	10,874 (91.6)	
Yes	7,567 (6.85)	5,165 (6.28)	1,400 (8.59)	1,002 (8.44)	
Neonatal jaundice, *n* (%)					<0.001
No	67,032 (60.7)	50,212 (61.0)	9,562 (58.6)	7,258 (61.1)	
Yes	43,415 (39.3)	32,054 (39.0)	6,743 (41.4)	4,618 (38.9)	
Neonatal respiratory distress syndrome, *n* (%)					<0.001
No	98,473 (89.2)	73,732 (89.6)	14,326 (87.9)	10,415 (87.7)	
Yes	11,974 (10.8)	8,534 (10.4)	1,979 (12.1)	1,461 (12.3)	

^a^ The prepandemic period is January 1, 2016, to March 12, 2020; the early pandemic period is March 13, 2020, to March 12, 2021; and the late pandemic period is March 13, 2021, to December 31, 2021.

^b^ *P* values were assessed using one-way analysis of variance (continuous outcome) or χ^2^ test (categorical outcome).

^c^ Other race and ethnicity includes Asian, Native American, and Hawaiian or Pacific Islander.

SD, standard deviation.

Compared with deliveries before the COVID-19 pandemic, the multivariable-adjusted (maternal age, race, and ethnicity) relative risks of adverse pregnancy outcomes among deliveries in the early pandemic and the late pandemic periods were significantly higher for primary cesarean section (OR: 1.11, 95% CI: 1.05–1.17; OR: 1.19, 95% CI: 1.12–1.27), LBW (OR: 1.13, 95% CI: 1.06–1.21; OR: 1.11, 95% CI: 1.03–1.20), SGA (OR: 1.82, 95% CI: 1.70–1.95; OR: 1.72, 95% CI: 1.59–1.87), LGA (OR: 1.30, 95% CI: 1.19–1.42; OR: 1.12, 95% CI: 1.00–1.24), macrosomia (OR: 1.28, 95% CI: 1.18–1.40; OR: 1.11, 95% CI: 1.00–1.23), neonatal hypoglycemia (OR: 1.39, 95% CI: 1.31–1.48; OR: 1.37, 95% CI: 1.28–1.47), neonatal jaundice (OR: 1.11, 95% CI: 1.07–1.15; OR: 0.99, 95% CI: 0.96–1.03), and NRDS (OR: 1.20, 95% CI: 1.14–1.27; OR: 1.23, 95% CI: 1.16–1.30), respectively (Table 2).

**Table 2.** Associations Between Diabetes During Pregnancy and Adverse Pregnancy Outcomes Stratified by the COVID-19 Pandemic^a^

Outcomes	Total (*N* = 110,447)	Odds ratios (95% confidence intervals)^b^			*p* for trend	*p* for interaction
		**Normal glucose** (*N* = 100**,180)**	**Gestational diabetes mellitus** (*N* = 7**,357)**	**Diabetes before pregnancy** (*N* = 2**,910)**		
Preterm birth (*N* = 8,343)		1.00	1.18 (1.08–1.28)	1.68 (1.50–1.88)	<0.001	0.07
Prepandemic	1.00	1.00	1.25 (1.13–1.38)	1.72 (1.51–1.96)	<0.001	
Early pandemic	0.92 (0.86–0.98)	0.92 (0.86–0.99)	1.08 (0.88–1.32)	1.41 (1.04–1.93)	0.005	
Late pandemic	0.97 (0.90–1.04)	1.00 (0.92–1.08)	0.80 (0.60–1.07)	1.58 (1.13–2.22)	0.27	
*p* for trend	0.07	0.35	0.003	0.40		
Primary cesarean section (*N* = 10,398)		1.00	1.36 (1.26–1.46)	2.26 (2.05–2.49)	<0.001	0.83
Prepandemic	1.00	1.00	1.36 (1.25–1.49)	2.24 (2.00–2.51)	<0.001	
Early pandemic	1.11 (1.05–1.17)	1.10 (1.04–1.17)	1.40 (1.19–1.65)	2.69 (2.13–3.39)	<0.001	
Late pandemic	1.19 (1.12–1.27)	1.19 (1.11–1.27)	1.69 (1.38–2.05)	2.56 (1.96–3.34)	<0.001	
*p* for trend	<0.001	<0.001	0.08	0.19		
Low birth weight (*N* = 7,303)		1.00	1.06 (0.96–1.16)	2.20 (1.97–2.45)	<0.001	0.30
Prepandemic	1.00	1.00	1.10 (0.98–1.24)	2.23 (1.97–2.53)	0.007	
Early pandemic	1.13 (1.06–1.21)	1.15 (1.07–1.23)	1.04 (0.84–1.30)	2.72 (2.10–3.52)	<0.001	
Late pandemic	1.11 (1.03–1.20)	1.13 (1.04–1.23)	1.14 (0.86–1.49)	2.01 (1.46–2.78)	<0.001	
*p* for trend	<0.001	<0.001	0.97	0.99		
Small for gestational age (*N* = 5,386)		1.00	0.84 (0.75–0.95)	1.12 (0.96–1.32)	0.66	0.33
Prepandemic	1.00	1.00	0.84 (0.72–0.98)	0.98 (0.79–1.21)	0.13	
Early pandemic	1.82 (1.70–1.95)	1.81 (1.69–1.95)	1.39 (1.10–1.77)	2.63 (1.93–3.57)	0.56	
Late pandemic	1.72 (1.59–1.87)	1.71 (1.58–1.86)	1.40 (1.04–1.89)	2.18 (1.50–3.17)	0.64	
*p* for trend	<0.001	<0.001	<0.001	<0.001		
Large for gestational age (*N* = 3,439)		1.00	1.42 (1.26–1.60)	3.05 (2.65–3.51)	<0.001	0.50
Prepandemic	1.00	1.00	1.51 (1.31–1.74)	3.19 (2.71–3.76)	<0.001	
Early pandemic	1.30 (1.19–1.42)	1.34 (1.21–1.48)	1.60 (1.23–2.08)	3.63 (2.60–5.06)	<0.001	
Late pandemic	1.12 (1.00–1.24)	1.14 (1.01–1.28)	1.43 (1.01–2.02)	3.14 (2.11–4.67)	<0.001	
*p* for trend	<0.001	<0.001	0.89	0.85		
Macrosomia (*N* = 3,594)		1.00	1.47 (1.30–1.65)	3.09 (2.70–3.55)	<0.001	0.50
Prepandemic	1.00	1.00	1.54 (1.34–1.76)	3.25 (2.77–3.81)	<0.001	
Early pandemic	1.28 (1.18–1.40)	1.32 (1.20–1.46)	1.61 (1.24–2.08)	3.47 (2.48–4.84)	<0.001	
Late pandemic	1.11 (1.00–1.23)	1.12 (1.00–1.26)	1.58 (1.14–2.18)	3.25 (2.21–4.77)	<0.001	
*p* for trend	<0.001	<0.001	0.84	0.89		
Neonatal hypoglycemia (*N* = 7,567)		1.00	13.1 (12.4–14.0)	23.7 (21.9–25.7)	<0.001	0.004
Prepandemic	1.00	1.00	12.3 (11.4–13.2)	23.4 (21.3–25.8)	<0.001	
Early pandemic	1.39 (1.31–1.48)	1.21 (1.10–1.32)	20.0 (17.8–22.4)	33.2 (27.3–40.3)	<0.001	
Late pandemic	1.37 (1.28–1.47)	1.38 (1.25–1.52)	17.9 (15.4–20.7)	29.8 (24.0–37.0)	<0.001	
*p* for trend	<0.001	<0.001	<0.001	0.002		
Neonatal jaundice (*N* = 43,415)		1.00	1.22 (1.16–1.28)	1.62 (1.51–1.75)	<0.001	0.86
Prepandemic	1.00	1.00	1.24 (1.17–1.31)	1.60 (1.47–1.74)	<0.001	
Early pandemic	1.11 (1.07–1.15)	1.11 (1.07–1.15)	1.30 (1.16–1.45)	1.88 (1.55–2.27)	<0.001	
Late pandemic	0.99 (0.96–1.03)	0.99 (0.95–1.04)	1.17 (1.02–1.35)	1.66 (1.34–2.06)	<0.001	
*p* for trend	0.04	0.51	0.81	0.40		
Neonatal respiratory distress syndrome (*N* = 11,974)		1.00	1.33 (1.24–1.43)	3.00 (2.75–3.27)	<0.001	0.46
Prepandemic	1.00	1.00	1.32 (1.21–1.44)	3.07 (2.77–3.39)	<0.001	
Early pandemic	1.20 (1.14–1.27)	1.20 (1.14–1.27)	1.50 (1.29–1.76)	3.72 (3.02–4.59)	<0.001	
Late pandemic	1.23 (1.16–1.30)	1.23 (1.15–1.31)	1.73 (1.43–2.09)	3.09 (2.41–3.95)	<0.001	
*p* for trend	<0.001	<0.001	0.006	0.55		

^a^ The prepandemic period is January 1, 2016, to March 12, 2020; the early pandemic period is March 13, 2020, to March 12, 2021; and the late pandemic period is March 13, 2021, to December 31, 2021.

^b^ All odds ratios (95% confidence intervals) were assessed using logistic regression and adjusted models account for maternal age and race and ethnicity.

### Associations between hyperglycemic disorders and adverse pregnancy outcomes across the COVID-19 pandemic

Women with GDM during pregnancy and women with diabetes before pregnancy were significantly associated with increased ORs of preterm birth (1.18 and 1.68, all *p* < 0.001), primary cesarean section (1.36 and 2.26, all *p* < 0.001), LGA (1.42 and 3.05, all *p* < 0.001), macrosomia (1.47 and 3.09, all *p* < 0.001), neonatal hypoglycemia (13.1 and 23.7, all *p* < 0.001), neonatal jaundice (1.22 and 1.62, all *p* < 0.001), and NRDS (1.33 and 3.07, all *p* < 0.001), respectively, compared with women with normal glucose tolerance during pregnancy (Table 2 and Supplementary Table S2).

When stratified analyses were utilized, the positive association of hyperglycemic disorders during pregnancy with the risks of primary cesarean section, LBW, LGA, macrosomia, neonatal hypoglycemia, neonatal jaundice, and NRDS before the COVID-19 pandemic was consistent with those during the early and late pandemic (Table 2).

### Associations between HDP and adverse pregnancy outcomes across the COVID-19 pandemic

Compared with normal blood pressure during pregnancy, gestational hypertension was associated with higher odds of primary cesarean section, SGA, LGA, macrosomia, neonatal hypoglycemia, neonatal jaundice, and NRDS and was associated with lower odds of preterm birth (all *p* < 0.05). Moreover, pre-eclampsia or eclampsia and pre-existing hypertension significantly elevated the odds of preterm birth, primary cesarean section, LBW, SGA, neonatal hypoglycemia, neonatal jaundice, and NRDS (all *p* < 0.05), respectively (Table 3 and Supplementary Table S3). Pre-existing hypertension was associated with increased odds of LGA and macrosomia (all *p* < 0.05).

**Table 3.** Associations Between Hypertensive Disorders of Pregnancy and Adverse Pregnancy Outcomes Stratified by the COVID-19 Pandemic^a^

Outcomes	Odds ratios (95% confidence intervals)^b^				*p* for trend	*p* for interaction
	**Normal blood pressure** (*N* = 86**,318)**	**Gestational hypertension (***N* = 7**,341)**	**Preeclampsia or eclampsia** (*N* = 8**,048)**	**Pre-existing hypertension **(*N* = 8**,740)**		
Preterm birth (*N* = 8,343)	1.00	0.78 (0.71–0.87)	1.49 (1.38–1.60)	1.37 (1.27–1.48)	<0.001	0.22
Prepandemic	1.00	0.79 (0.70–0.89)	1.55 (1.41–1.69)	1.41 (1.29–1.54)	<0.001	
Early pandemic	0.92 (0.85–0.99)	0.80 (0.63–1.01)	1.24 (1.03–1.49)	1.31 (1.09–1.57)	<0.001	
Late pandemic	1.01 (0.93–1.10)	0.66 (0.49–0.88)	1.39 (1.14–1.70)	1.09 (0.86–1.36)	0.12	
*p* for trend	0.50	0.34	0.09	0.03		
Primary cesarean section (*N* = 10,398)	1.00	1.24 (1.15–1.34)	1.56 (1.46–1.68)	1.85 (1.73–1.97)	<0.001	0.70
Prepandemic	1.00	1.18 (1.07–1.30)	1.58 (1.45–1.72)	1.82 (1.69–1.96)	<0.001	
Early pandemic	1.08 (1.01–1.16)	1.47 (1.24–1.75)	1.57 (1.33–1.84)	2.06 (1.77–2.39)	<0.001	
Late pandemic	1.15 (1.07–1.25)	1.53 (1.26–1.85)	1.77 (1.48–2.10)	2.22 (1.88–2.62)	<0.001	
*p* for trend	<0.001	0.004	0.40	0.01		
Low birth weight (*N* = 7,303)	1.00	0.96 (0.85–1.08)	4.89 (4.59–5.21)	3.54 (3.31–3.79)	<0.001	0.77
Prepandemic	1.00	0.93 (0.81–1.07)	4.94 (4.58–5.34)	3.59 (3.32–3.88)	<0.001	
Early pandemic	1.10 (1.00–1.20)	1.14 (0.89–1.46)	4.93 (4.30–5.66)	3.90 (3.37–4.53)	<0.001	
Late pandemic	1.06 (0.95–1.18)	1.00 (0.74–1.35)	5.29 (4.55–6.17)	3.37 (2.82–4.02)	<0.001	
*p* for trend	0.07	0.41	0.49	0.81		
Small for gestational age (*N* = 5,386)	1.00	1.63 (1.48–1.80)	2.40 (2.21–2.60)	1.68 (1.54–1.84)	<0.001	0.06
Prepandemic	1.00	1.56 (1.37–1.77)	2.47 (2.23–2.74)	1.64 (1.46–1.83)	<0.001	
Early pandemic	1.87 (1.72–2.03)	2.67 (2.19–3.26)	3.63 (3.08–4.29)	2.74 (2.27–3.30)	<0.001	
Late pandemic	1.60 (1.44–1.77)	2.94 (2.37–3.65)	3.68 (3.05–4.45)	3.32 (2.72–4.05)	<0.001	
*p* for trend	<0.001	<0.001	<0.001	<0.001		
Large for gestational age (*N* = 3,439)	1.00	1.18 (1.03–1.35)	1.07 (0.94–1.22)	1.35 (1.20–1.52)	<0.001	0.14
Prepandemic	1.00	1.13 (0.96–1.33)	1.03 (0.87–1.21)	1.44 (1.26–1.66)	<0.001	
Early pandemic	1.32 (1.19–1.47)	1.75 (1.35–2.27)	1.20 (0.89–1.63)	1.47 (1.11–1.94)	0.49	
Late pandemic	1.10 (0.97–1.25)	1.22 (0.86–1.73)	1.56 (1.15–2.10)	1.24 (0.88–1.76)	0.12	
*p* for trend	<0.001	0.14	0.01	0.50		
Macrosomia (*N* = 3,594)	1.00	1.19 (1.05–1.36)	1.05 (0.92–1.20)	1.34 (1.19–1.50)	<0.001	0.12
Prepandemic	1.00	1.15 (0.99–1.35)	1.00 (0.85–1.18)	1.44 (1.26–1.65)	<0.001	
Early pandemic	1.31 (1.18–1.45)	1.67 (1.28–2.16)	1.17 (0.87–1.58)	1.45 (1.10–1.91)	0.52	
Late pandemic	1.09 (0.96–1.24)	1.27 (0.91–1.77)	1.55 (1.16–2.08)	1.18 (0.84–1.67)	0.16	
*p* for trend	0.001	0.17	0.007	0.36		
Neonatal hypoglycemia (*N* = 7,567)	1.00	1.55 (1.41–1.69)	2.63 (2.45–2.83)	3.45 (3.24–3.68)	<0.001	0.29
Prepandemic	1.00	1.60 (1.43–1.79)	2.62 (2.39–2.86)	3.53 (3.27–3.81)	<0.001	
Early pandemic	1.42 (1.31–1.53)	2.05 (1.69–2.48)	3.21 (2.75–3.75)	4.48 (3.89–5.15)	<0.001	
Late pandemic	1.33 (1.21–1.46)	1.71 (1.35–2.16)	3.79 (3.21–4.48)	4.48 (3.82–5.26)	<0.001	
*p* for trend	<0.001	0.18	<0.001	<0.001		
Neonatal jaundice (*N* = 43,415)	1.00	1.19 (1.13–1.25)	1.57 (1.50–1.65)	1.45 (1.39–1.52)	<0.001	0.09
Prepandemic	1.00	1.20 (1.13–1.27)	1.53 (1.45–1.62)	1.40 (1.33–1.48)	<0.001	
Early pandemic	1.08 (1.04–1.12)	1.27 (1.14–1.42)	1.74 (1.57–1.94)	1.72 (1.54–1.92)	<0.001	
Late pandemic	0.96 (0.92–1.01)	1.08 (0.95–1.23)	1.67 (1.48–1.88)	1.52 (1.34–1.72)	<0.001	
*p* for trend	0.995	0.41	0.05	0.02		
Neonatal respiratory distress syndrome (*N* = 11,974)	1.00	1.19 (1.10–1.29)	2.74 (2.59–2.91)	2.64 (2.50–2.80)	<0.001	0.52
Prepandemic	1.00	1.22 (1.11–1.34)	2.72 (2.53–2.92)	2.71 (2.53–2.90)	<0.001	
Early pandemic	1.20 (1.12–1.28)	1.39 (1.17–1.65)	3.07 (2.70–3.49)	2.97 (2.60–3.38)	<0.001	
Late pandemic	1.20 (1.12–1.30)	1.22 (0.99–1.50)	3.45 (3.00–3.98)	2.93 (2.51–3.41)	<0.001	
*p* for trend	<0.001	0.60	0.001	0.22		

^a^ The prepandemic period is January 1, 2016, to March 12, 2020; the early pandemic period is March 13, 2020, to March 12, 2021; and the late pandemic period is March 13, 2021, to December 31, 2021.

^b^ All odds ratios (95% confidence intervals) were assessed using logistic regression and adjusted models account for maternal age and race and ethnicity.

When stratified analyses were utilized, the positive associations of gestational hypertension, pre-eclampsia or eclampsia and pre-existing hypertension with the risks of primary cesarean section, LBW, SGA, neonatal hypoglycemia, neonatal jaundice, and NRDS remained consistent mostly before the COVID-19 pandemic and during the early and late pandemic (Table 3). The associations of pre-existing hypertension with the risks of LGA and macrosomia were consistent before the COVID-19 pandemic and during the early and late pandemic.

### Joint associations of hyperglycemic disorders and HDP with adverse pregnancy outcomes

When we assessed the joint associations of hyperglycemic disorders during pregnancy and HDP with adverse pregnancy outcomes, women were divided into four groups based on results from Tables 2 and 3: women with normal glucose and blood pressure during pregnancy, women with hyperglycemic disorders during pregnancy only (GDM during pregnancy or diabetes before pregnancy), HDP only (gestational hypertension, pre-eclampsia, eclampsia, or pre-existing chronic hypertension), and both hyperglycemic disorders during pregnancy and HDP (Table 4 and Supplementary Table S4). In comparison with normal glucose and blood pressure during pregnancy, hyperglycemic disorders during pregnancy were only associated with the higher risks of primary cesarean section (OR: 1.31, 95% CI: 1.19–1.44), LGA (OR: 1.26, 95% CI: 1.08–1.47), macrosomia (OR: 1.29, 95% CI: 1.11–1.49), neonatal hypoglycemia (OR: 15.4, 95% CI: 14.3–16.6), neonatal jaundice (OR: 1.16, 95% CI: 1.09–1.23), and NRDS (OR: 1.20, 95% CI: 1.09–1.33); HDP only was associated with the higher risks of preterm birth (OR: 1.16, 95% CI: 1.10–1.23), primary cesarean section (OR 1.50, 95% CI 1.43–1.58), LBW (OR: 3.05, 95% CI: 2.89–3.21), SGA (OR: 1.97, 95% CI: 1.86–2.10), neonatal hypoglycemia (OR: 2.33, 95% CI: 2.17–2.50), neonatal jaundice (OR: 1.36, 95% CI: 1.32–1.41), and NRDS (OR: 2.08, 95% CI: 1.99–2.18); the presence of both hyperglycemic disorders during pregnancy and HDP was associated with the highest risks of above all adverse pregnancy outcomes (all *p* for trend <0.001).

**Table 4.** Associations of the Status of Diabetes and Hypertensive Disorders of Pregnancy with Adverse Pregnancy Outcomes

Outcomes	Odds ratios (95% confidence intervals)^a^				*p* for trend	*p* for interaction
	**Normal glucose and blood pressure during pregnancy**	**Hyperglycemic disorders during pregnancy only**	**HDP only**	**Both hyperglycemic disorders during pregnancy and HDP**		
Preterm birth (*N* = 8,343)	1.00	1.10 (0.98–1.22)	1.16 (1.10–1.23)	1.61 (1.47–1.77)	<0.001	0.01
Primary cesarean section (*N* = 10,398)	1.00	1.31 (1.19–1.44)	1.50 (1.43–1.58)	2.21 (2.05–2.39)	<0.001	0.90
Low birth weight (*N* = 7,303)	1.00	0.89 (0.77–1.03)	3.05 (2.89–3.21)	2.96 (2.71–3.22)	<0.001	0.17
Small for gestational age (*N* = 5,386)	1.00	0.85 (0.72–1.00)	1.97 (1.86–2.10)	1.35 (1.19–1.52)	<0.001	0.11
Large for gestational age (*N* = 3,439)	1.00	1.26 (1.08–1.47)	1.03 (0.94–1.14)	2.47 (2.20–2.78)	<0.001	<0.001
Macrosomia (*N* = 3,594)	1.00	1.29 (1.11–1.49)	1.02 (0.93–1.12)	2.52 (2.25–2.82)	<0.001	<0.001
Neonatal hypoglycemia (*N* = 7,567)	1.00	15.4 (14.3–16.6)	2.33 (2.17–2.50)	24.7 (23.1–26.5)	<0.001	<0.001
Neonatal jaundice (*N* = 43,415)	1.00	1.16 (1.09–1.23)	1.36 (1.32–1.41)	1.68 (1.59–1.78)	<0.001	0.53
Neonatal respiratory distress syndrome (*N* = 11,974)	1.00	1.20 (1.09–1.33)	2.08 (1.99–2.18)	3.10 (2.89–3.32)	<0.001	0.18

^a^ All odds ratios (95% confidence intervals) were assessed using logistic regression and adjusted models account for maternal age and race and ethnicity.

HDP, hypertensive disorders of pregnancy.

## Discussion

In this Louisiana surveillance study, delivery during the early and late COVID-19 pandemic was associated with significantly higher risks of most adverse pregnancy outcomes. Both hyperglycemic disorders during pregnancy and HDP were associated with greater risks of most adverse pregnancy outcomes, and most of these associations were consistent before the COVID-19 pandemic and during the early and late pandemic.

There is accumulating evidence that COVID-19 infection during pregnancy is linked to adverse maternal–perinatal outcomes. A multinational cohort study across 18 countries revealed that pregnancies affected by COVID-19 faced increased risks of preeclampsia and preterm birth compared with those without infection.^16^ A systematic review and meta-analysis showed increased risks of maternal death, cesarean section, preterm birth, stillbirth, and admission to the neonatal intensive care unit among COVID-19-infected women.^17^ In addition to the direct impact of COVID-19 infection on pregnancy outcomes, the effect of the COVID-19 pandemic on pregnancy outcomes even among those without infection was also reported. A global systematic review observed the increased rate of ruptured ectopic pregnancies, stillbirths, and maternal deaths during the pandemic than before the pandemic.^18^ On the contrary, several studies showed an overall decline in the rate of preterm birth during the pandemic period,^19–21^ which was consistent with our findings. That might be due to differences in enforcement of lockdown orders during the pandemic, population heterogeneity, access to health care, and societal stressors.^22^ These findings were intriguing and might potentially provide the clues to solving the long-standing challenges in preventing preterm birth.

Our findings are in agreement with other studies on the association between hyperglycemic disorders during pregnancy and adverse maternal–perinatal outcomes.^23^ Since the onset of the COVID-19 pandemic, there is a growing interest in the association between GDM and COVID-19. Studies have shown that COVID-19 infection might increase the severity of GDM or promote the *de novo* onset of GDM by causing acute β-cell dysfunction, which led to increased prevalence of GDM and even adverse maternal–perinatal outcomes.^24–26^ However, comprehensive data on the combined effect of these conditions on pregnancy outcomes remain scarce. In the present study, we observed that only the association between hyperglycemic disorders and preterm birth varied across COVID-19 pandemic periods; however, GDM was not significantly increasing the risk of preterm birth during the late pandemic, contrary to overall population trends. This discrepancy may be explained by factors such as higher gestational weight gain during the pandemic, treatment differences (metformin or insulin), and other pregnancy complications.^27,28^ Further research is needed to confirm our findings and uncover the mechanisms underlying these differential associations.

The present study also indicated that HDP was associated with an increased risk of adverse pregnancy outcomes. Interestingly, we observed a paradoxical negative statistical association between gestational hypertension and preterm birth in this study, contrasting with another study that found no significant association between gestational hypertension and preterm birth.^29^ In addition, we noted that an increased risk of preterm birth, LGA, and macrosomia associated with HDP during the early and late COVID-19 pandemic was lower than the risk observed in the prepandemic period. These variations may be attributed to a pre-eclampsia-like syndrome induced by COVID-19, which can cause endothelial damage and alterations in angiotensin-converting enzyme 2-related pathways, similar to the changes seen in preeclampsia.^30,31^ Furthermore, other pertinent factors not considered in the present study, such as history of preterm delivery, parity, maternal body mass index, and additional pregnancy complications, should also be taken into account.^32^ These findings underscore the importance of using representative and diverse samples with comprehensive data and highlight the necessity for further research in this area.

## Strengths and Limitations

This study’s strengths include its large and diverse samples from a southern state, providing a significant breadth of data. Moreover, this study highlights findings from an underserved population in the United States, offering valuable insights for public health and policymakers.

Several limitations should be considered. First, adverse pregnancy outcomes using Medicaid claims data were limited by its nature as a claims database rather than a research database. A study examined preeclampsia in the U.S. Medicaid Analytical Extraction and found that the positive predictive value of preeclampsia was 67% when cross-checked with hospital records.^33^ Moreover, other studies have demonstrated high validity of Medicaid data in research.^34,35^ In the present analysis, we have no data to verify the diagnosis and rely on the accuracy of the claims. Second, due to its observational design and reliance on administrative data, the present study cannot ascertain the mechanisms driving the observed differences in the associations of maternal hyperglycemic disorders during pregnancy and HDP with adverse pregnancy outcomes throughout the COVID-19 pandemic periods. Third, while the study samples were representative of a metropolitan area, the findings may not extend to other regions, limiting generalizability. Fourth, the analysis lacked data on several important factors such as parity, maternal body mass index, gestational weight gain, and other pregnancy-related conditions, which were crucial confounders that could influence outcomes. Lastly, the study’s scope was confined to the initial 2 years of the COVID-19 pandemic, leaving the long-term effects on these associations undetermined.

## Conclusions

The risks of adverse maternal and neonatal outcomes significantly increased during the early and late stages of the COVID-19 pandemic. Both hyperglycemic disorders during pregnancy and HDP were associated with greater risks of most adverse pregnancy outcomes, and most of these associations were consistent before the COVID-19 pandemic and during the early and late pandemic.

## Data Availability

The data are not publicly available. Data can be accessed from the principal investigator through formal request for research purposes. Please contact gang.hu@pbrc.edu to seek approval for data access.

## Abbreviations Used

CI: Confidence interval
COVID-19: Coronavirus disease 2019
GDM: Maternal gestational diabetes mellitus
HDP: Hypertensive disorders of pregnancy
ICD-10: Tenth Revisions of the International Classification of Disease
LBW: Low birth weight
LGA: Large for gestational age
NRDS: Neonatal respiratory distress syndrome
OR: Odds ratio
SGA: Small for gestational age
