# Supplementary material for: Impact of Maternal Hyperglycemic and Hypertensive Disorders on Perinatal Outcomes Across the COVID-19 Pandemic
Source: Womens Health Rep (New Rochelle). 2025 Apr 28;6(1):504–14. doi: 10.1089/whr.2025.0019 (PMC12177329; doi:10.1089/whr.2025.0019)
Supplement: Supplementary Table S1 [file whr.2025.0019_supplementary_table_s1.docx]

**Supplemental Table 1.** Numbers of participates in subgroups by covid pandemic stratified by diabetes and hypertension during pregnancy^a^.

|  | **Pre-pandemic** | **Early pandemic** | **Late pandemic** |
| --- | --- | --- | --- |
|  | **(N=82,266)** | **(N=16,305)** | **(N=11,876)** |
| Diabetes during pregnancy |  |  |  |
| Normal | 74,934 | 14,537 | 10,709 |
| Gestational diabetes mellitus | 5,191 | 1,338 | 828 |
| Diabetes before pregnancy | 2,141 | 430 | 339 |
| Hypertensive disorders of pregnancy |  |  |  |
| Normal | 65,237 | 12,283 | 8,798 |
| Gestational hypertension | 5,083 | 1,275 | 983 |
| Preeclampsia or eclampsia | 5,558 | 1,399 | 1,091 |
| Pre-existing hypertension | 6,388 | 1,348 | 1,004 |

^a^ The pre-pandemic period is January 1, 2016, to March 12, 2020; the early pandemic period is March 13, 2020, to March 12, 2021; and the late pandemic period is March 13, 2021, to December 31, 2021.
