# Supplementary material for: Impact of Maternal Hyperglycemic and Hypertensive Disorders on Perinatal Outcomes Across the COVID-19 Pandemic
Source: Womens Health Rep (New Rochelle). 2025 Apr 28;6(1):504–14. doi: 10.1089/whr.2025.0019 (PMC12177329; doi:10.1089/whr.2025.0019)
Supplement: Supplementary Table S2 [file whr.2025.0019_supplementary_table_s2.docx]

**Supplemental Table 2**. Characteristics and adverse pregnancy outcomes of participants by diabetes during pregnancy.

| **Variables** | **Normal glucose** | **Gestational diabetes mellitus** | **Diabetes before pregnancy** | ***P* value^a^** |
| --- | --- | --- | --- | --- |
| No. of participants | 100,180 | 7,357 | 2,910 |  |
| Age, mean (SD), years | 25.8 (5.88) | 29.7 (6.29) | 30.3 (6.52) | <0.001 |
| Race and ethnicity, n (%)^b^ |  |  |  | <0.001 |
| White | 43,912 (43.8) | 3,432 (46.6) | 1,041 (35.8) |  |
| Black | 40,518 (40.4) | 2,293 (31.2) | 1,444 (49.6) |  |
| Other or unknown | 15,750 (15.7) | 1,632 (22.2) | 425 (14.6) |  |
| Preterm birth, n (%) |  |  |  | <0.001 |
| No | 92,807 (92.6) | 6,739 (91.6) | 2,558 (87.9) |  |
| Yes | 7,373 (7.36) | 618 (8.40) | 352 (12.1) |  |
| Primary caesarean section, n (%) |  |  |  | <0.001 |
| No | 91,234 (91.1) | 6,460 (87.8) | 2,355 (80.9) |  |
| Yes | 8,946 (8.93) | 897 (12.2) | 555 (19.1) |  |
| Low birth weight, n (%) |  |  |  | <0.001 |
| No | 93,791 (93.6) | 6,864 (93.3) | 2,489 (85.5) |  |
| Yes | 6,389 (6.38) | 493 (6.70) | 421 (14.5) |  |
| Small for gestational age, n (%) |  |  |  | <0.001 |
| No | 95,264 (95.1) | 7,059 (95.9) | 2,738 (94.1) |  |
| Yes | 4,916 (4.91) | 298 (4.05) | 172 (5.91) |  |
| Large for gestational age, n (%) |  |  |  | <0.001 |
| No | 97,298 (97.1) | 7,037 (95.7) | 2,673 (91.9) |  |
| Yes | 2,882 (2.88) | 320 (4.35) | 237 (8.14) |  |
| Macrosomia, n (%) |  |  |  | <0.001 |
| No | 97,177 (97.0) | 7,015 (95.4) | 2,661 (91.4) |  |
| Yes | 3,003 (3.00) | 342 (4.65) | 249 (8.56) |  |
| Neonatal hypoglycemia, n (%) |  |  |  | <0.001 |
| No | 96,521 (96.3) | 4,856 (66.0) | 1,503 (51.6) |  |
| Yes | 3,659 (3.65) | 2,501 (34.0) | 1,407 (48.4) |  |
| Neonatal jaundice, n (%) |  |  |  | <0.001 |
| No | 61,465 (61.4) | 4,102 (55.8) | 1,465 (50.3) |  |
| Yes | 38,715 (38.6) | 3,255 (44.2) | 1,445 (49.7) |  |
| Neonatal respiratory distress syndrome, n (%) |  |  |  | <0.001 |
| No | 89,952 (89.8) | 6,384 (86.8) | 2,137 (73.4) |  |
| Yes | 10,228 (10.2) | 973 (13.2) | 773 (26.6) |  |

^a^ *P* values were assessed using one-way ANOVA (continuous outcome) or χ^2^ test (categorical outcome).

^b^ Other race and ethnicity includes Asian, Native American, and Hawaiian or Pacific Islander.
