# Supplementary material for: Impact of Maternal Hyperglycemic and Hypertensive Disorders on Perinatal Outcomes Across the COVID-19 Pandemic
Source: Womens Health Rep (New Rochelle). 2025 Apr 28;6(1):504–14. doi: 10.1089/whr.2025.0019 (PMC12177329; doi:10.1089/whr.2025.0019)
Supplement: Supplementary Table S3 [file whr.2025.0019_supplementary_table_s3.docx]

**Supplemental Table 3**. Characteristics and adverse pregnancy outcomes of participants by hypertensive disorders of pregnancy.

| **Variables** | **Normal blood pressure** | **Gestational hypertension** | **Preeclampsia or eclampsia** | **Pre-existing hypertension** | ***P* value^a^** |
| --- | --- | --- | --- | --- | --- |
| No. of participants | 86,318 | 7,341 | 8,048 | 8,740 |  |
| Age, mean (SD), years | 25.9 (5.86) | 25.8 (6.04) | 25.3 (6.13) | 29.9 (6.49) | <0.001 |
| Race and ethnicity, n (%)^b^ |  |  |  |  | <0.001 |
| White | 38,854 (45.0%) | 3,391 (46.2%) | 3,110 (38.6%) | 3,030 (34.7%) |  |
| Black | 32,422 (37.6%) | 3,112 (42.4%) | 3,868 (48.1%) | 4,853 (55.5%) |  |
| Other or unknown | 15,042 (17.4%) | 838 (11.4%) | 1,070 (13.3%) | 857 (9.81%) |  |
| Preterm birth, n (%) |  |  |  |  | <0.001 |
| No | 80,126 (92.8%) | 6,917 (94.2%) | 7,199 (89.5%) | 7,862 (90.0%) |  |
| Yes | 6,192 (7.17%) | 424 (5.78%) | 849 (10.5%) | 878 (10.0%) |  |
| Primary caesarean section, n (%) |  |  |  |  | <0.001 |
| No | 79,019 (91.5%) | 6,586 (89.7%) | 7,032 (87.4%) | 7,412 (84.8%) |  |
| Yes | 7,299 (8.46%) | 755 (10.3%) | 1,016 (12.6%) | 1,328 (15.2%) |  |
| Low birth weight, n (%) |  |  |  |  | <0.001 |
| No | 82,352 (95.4%) | 7,008 (95.5%) | 6,457 (80.2%) | 7,327 (83.8%) |  |
| Yes | 3,966 (4.59%) | 333 (4.54%) | 1,591 (19.8%) | 1,413 (16.2%) |  |
| Small for gestational age, n (%) |  |  |  |  | <0.001 |
| No | 82,817 (95.9%) | 6,862 (93.5%) | 7,274 (90.4%) | 8,108 (92.8%) |  |
| Yes | 3,501 (4.06%) | 479 (6.52%) | 774 (9.62%) | 632 (7.23%) |  |
| Large for gestational age, n (%) |  |  |  |  | <0.001 |
| No | 83,712 (97.0%) | 7,087 (96.5%) | 7,802 (96.9%) | 8,407 (96.2%) |  |
| Yes | 2,606 (3.02%) | 254 (3.46%) | 246 (3.06%) | 333 (3.81%) |  |
| Macrosomia, n (%) |  |  |  |  | <0.001 |
| No | 83,592 (96.8%) | 7,072 (96.3%) | 7,795 (96.9%) | 8,394 (96.0%) |  |
| Yes | 2,726 (3.16%) | 269 (3.66%) | 253 (3.14%) | 346 (3.96%) |  |
| Neonatal hypoglycemia, n (%) |  |  |  |  | <0.001 |
| No | 81,856 (94.8%) | 6,767 (92.2%) | 7,037 (87.4%) | 7,220 (82.6%) |  |
| Yes | 4,462 (5.17%) | 574 (7.82%) | 1,011 (12.6%) | 1,520 (17.4%) |  |
| Neonatal jaundice, n (%) |  |  |  |  | <0.001 |
| No | 53,719 (62.2%) | 4,302 (58.6%) | 4,206 (52.3%) | 4,805 (55.0%) |  |
| Yes | 32,599 (37.8%) | 3,039 (41.4%) | 3,842 (47.7%) | 3,935 (45.0%) |  |
| Neonatal respiratory distress syndrome, n (%) |  |  |  |  | <0.001 |
| No | 78,694 (91.2%) | 6,569 (89.5%) | 6,339 (78.8%) | 6,871 (78.6%) |  |
| Yes | 7,624 (8.83%) | 772 (10.5%) | 1,709 (21.2%) | 1,869 (21.4%) |  |

^a^ *P* values were assessed using one-way ANOVA (continuous outcome) or χ^2^ test (categorical outcome).

^b^ Other race and ethnicity includes Asian, Native American, and Hawaiian or Pacific Islander.
