# Supplementary material for: Impact of Maternal Hyperglycemic and Hypertensive Disorders on Perinatal Outcomes Across the COVID-19 Pandemic
Source: Womens Health Rep (New Rochelle). 2025 Apr 28;6(1):504–14. doi: 10.1089/whr.2025.0019 (PMC12177329; doi:10.1089/whr.2025.0019)
Supplement: Supplementary Table S4 [file whr.2025.0019_supplementary_table_s4.docx]

**Supplemental Table 4**. Characteristics and adverse pregnancy outcomes of participants by hypertensive disorders of pregnancy and diabetes during pregnancy.

| **Variables** | **Normal glucose and blood pressure during pregnancy** | **Hyperglycemic disorders during pregnancy only** | **HDP only** | **Both hyperglycemic disorders during pregnancy and HDP** | ***P* value^a^** |
| --- | --- | --- | --- | --- | --- |
| No. of participants | 80,129 | 4,909 | 20,051 | 5,358 |  |
| Age, mean (SD), years | 25.6 (5.73) | 29.5 (6.19) | 26.4 (6.37) | 30.2 (6.48) | <0.001 |
| Race and ethnicity, n (%)^b^ |  |  |  |  | <0.001 |
| White | 36,026 (45.0%) | 2,340 (47.7%) | 7,886 (39.3%) | 2,133 (39.8%) |  |
| Black | 30,584 (38.2%) | 1,289 (26.3%) | 9,934 (49.5%) | 2,448 (45.7%) |  |
| Other or unknown | 13,519 (16.9%) | 1,280 (26.1%) | 2,231 (11.1%) | 777 (14.5%) |  |
| Preterm birth, n (%) |  |  |  |  | <0.001 |
| No | 74,442 (92.9%) | 4,540 (92.5%) | 18,365 (91.6%) | 4,757 (88.8%) |  |
| Yes | 5,687 (7.10%) | 369 (7.52%) | 1,686 (8.41%) | 601 (11.2%) |  |
| Primary caesarean section, n (%) |  |  |  |  | <0.001 |
| No | 73,579 (91.8%) | 4,377 (89.2%) | 17,655 (88.1%) | 4,438 (82.8%) |  |
| Yes | 6,550 (8.17%) | 532 (10.8%) | 2,396 (11.9%) | 920 (17.2%) |  |
| Low birth weight, n (%) |  |  |  |  | <0.001 |
| No | 76,455 (95.4%) | 4,713 (96.0%) | 17,336 (86.5%) | 4,640 (86.6%) |  |
| Yes | 3,674 (4.59%) | 196 (3.99%) | 2,715 (13.5%) | 718 (13.4%) |  |
| Small for gestational age, n (%) |  |  |  |  | <0.001 |
| No | 76,848 (95.9%) | 4,744 (96.6%) | 18,416 (91.8%) | 5,053 (94.3%) |  |
| Yes | 3,281 (4.09%) | 165 (3.36%) | 1,635 (8.15%) | 305 (5.69%) |  |
| Large for gestational age, n (%) |  |  |  |  | <0.001 |
| No | 77,815 (97.1%) | 4,716 (96.1%) | 19,483 (97.2%) | 4,994 (93.2%) |  |
| Yes | 2,314 (2.89%) | 193 (3.93%) | 568 (2.83%) | 364 (6.79%) |  |
| Macrosomia, n (%) |  |  |  |  | <0.001 |
| No | 77,713 (97.0%) | 4,704 (95.8%) | 19,464 (97.1%) | 4,972 (92.8%) |  |
| Yes | 2,416 (3.02%) | 205 (4.18%) | 587 (2.93%) | 386 (7.20%) |  |
| Neonatal hypoglycemia, n (%) |  |  |  |  | <0.001 |
| No | 77,793 (97.1%) | 3,330 (67.8%) | 18,728 (93.4%) | 3,029 (56.5%) |  |
| Yes | 2,336 (2.92%) | 1,579 (32.2%) | 1,323 (6.60%) | 2,329 (43.5%) |  |
| Neonatal jaundice, n (%) |  |  |  |  | <0.001 |
| No | 50,185 (62.6%) | 2,850 (58.1%) | 11,280 (56.3%) | 2,717 (50.7%) |  |
| Yes | 29,944 (37.4%) | 2,059 (41.9%) | 8,771 (43.7%) | 2,641 (49.3%) |  |
| Neonatal respiratory distress syndrome, n (%) |  |  |  |  | <0.001 |
| No | 73,259 (91.4%) | 4,413 (89.9%) | 16,693 (83.3%) | 4,108 (76.7%) |  |
| Yes | 6,870 (8.57%) | 496 (10.1%) | 3,358 (16.7%) | 1,250 (23.3%) |  |

Abbreviations: HDP, hypertensive disorders of pregnancy.

^a^ *P* values were assessed using one-way ANOVA (continuous outcome) or χ^2^ test (categorical outcome).

^b^ Other race and ethnicity includes Asian, Native American, and Hawaiian or Pacific Islander.
